# Virtual reality and 3D printing in head and neck cancer: an educational experience

**DOI:** 10.3389/fonc.2025.1695870

**Published:** 2026-01-08

**Authors:** Fabrizio Ferretti, Francesca Nonis, Andrea Novaresio, Enrica Panico, Emanuele Zavattero, Claudia Borbon, Sandro Moos, Enrico Vezzetti, Massimo Fasolis, Guglielmo Ramieri

**Affiliations:** 1 Department of Surgical Sciences, Division of Maxillofacial Surgery, University of Turin, Città della Salute e della Scienza Hospital, Torino, Italy; 2 Department of Management and Production Engineering, Politecnico di Torino, Torino, Italy

**Keywords:** maxillofacial surgery, virtual reality, three-dimensional printing, mandibular reconstruction, fibula free flap, surgical education, user-centered design

## Abstract

**Background:**

In recent years, the growing adoption of Virtual Reality (VR) and 3D printing technologies has revolutionized surgical training by providing innovative opportunities for hands-on education. This study investigates the combined use of VR and 3D printed personalized anatomical models and cutting guides within the field of oral and maxillofacial oncologic surgery.

Materials and methods: A mandibular tumour case was developed using the proposed approach, integrating both virtual and physical tools. Feedback was gathered from twelve surgical residents regarding their understanding of the case, the effectiveness of the immersive and three-dimensional technologies, and their overall satisfaction with the training experience.

**Results:**

Participants reported enhanced comprehension of complex surgical scenarios and valued the practical utility of the VR simulator combined with 3D printed models. The immersive environment facilitated skill acquisition in a risk-free setting.

**Conclusion:**

The findings underscore the significant added value of integrating VR and 3D printing technologies in surgical training, preparation, and simulation. This approach offers a safe, effective training platform that improves readiness for complex procedures in oncologic surgery and has the potential to be extended to other branches of maxillofacial surgery.

## Introduction

The intricate anatomy of the head and neck region and the complexity associated to surgical procedures provide fertile ground for the integration of advanced technologies. Among these, Virtual Reality (VR) is emerging as an effective and intuitive tool for surgical planning (1), while three-dimensional (3D) modelling and printing technologies allow surgeons to conduct detailed and independent preoperative planning (2). Together, these technologies enhance the understanding and visualization of pathology, improve assessment of operability, and support individualized treatment strategies.

VR enables the creation of immersive, interactive environments for exploring anatomical regions, offering valuable applications in diagnosis, surgical planning, and training. Its use extends across multiple domains, from dental implantology and orthognathic surgery to complex 3D planning for post-tumour mandibular reconstructions and fracture repair, improving precision and safety through predictive planning and intraoperative navigation. In addition, VR provides a powerful educational platform, allowing standardized yet flexible learning experiences where trainees can visualize and practice surgical procedures in a controlled and replicable way (3, 4).

Similarly, 3D printing has transformed modern healthcare through the development of patient-specific models and implants, representing a key step toward precision medicine. In oral and maxillofacial surgery, its most relevant applications include traumatology, orthognathic surgery, and joint reconstruction, where custom-made solutions have led to improved surgical accuracy and clinical outcomes (5). Numerous studies have underscored the importance of these technologies in mandibular reconstruction following tumour resection (6).

In oncologic surgery, surgical precision, from preoperative planning to intraoperative execution, is crucial. This accuracy depends on the surgeon’s ability to spatially perceive the lesion and its anatomical relationships. While conventional computed tomography (CT) provides essential diagnostic data, it offers only a two-dimensional representation, limiting three-dimensional comprehension.

This study aims to evaluate the application of VR and 3D printing in head and neck oncologic surgery as part of an educational and training experience for young maxillofacial residents. The initiative is designed with a didactic purpose, demonstrating and explaining the decision-making process involved in surgical design and planning. Using a VR headset, specifically the Meta Quest Pro, participants non-experts in the procedure planned a mandibular resection and reconstruction with fibula flap harvesting. The same procedure was carried out on 3D printed models. Finally, participants completed a questionnaire to assess their experience. From an educational standpoint, this pilot experience is grounded in experiential learning theory of Dale’s Cone of Experience (7), which supports the use of immersive, practice-oriented activities to improve comprehension and skill acquisition.

This scalable and extendable approach may serve as a model for future training programs in complex head and neck surgery.

## Materials and methods

Before starting the simulation, participants received a brief orientation session on how to use the VR environment, and the 3D printed cutting guides and anatomical models. No specific theoretical discussion of the surgical procedure was provided at this stage. After completing both practical sessions, participants engaged in a debriefing with an expert surgeon, during which the underlying surgical planning principles were explained and discussed.

Data collection aimed to assess the feasibility, usability, and educational potential of the proposed workflow. Specifically, procedure duration, accuracy in tumour localization and resection planning, and correct placement of osteotomy guides were measured to estimate the overall practicality and usability of the experience.

### Participants

Twelve maxillofacial surgery residents (ten female), evenly split between first-, second- and third-year trainees, participated in a hands-on educational session that combined a VR surgical simulator with 3D printed anatomical models of a mandibular malignant tumour. Upon entering the VR environment, participants were presented with an introductory panel that guided them through the different phases of the simulation. The same surgical workflow performed in VR was then replicated on stereolithographic models, which included a fibula, a mandible with the tumour *in situ*, and the corresponding cutting and repositioning guides for flap insertion.

For each participant, the following parameters were recorded: total time required to complete the exercise; the axial, sagittal, and coronal CT section plane best identifying the tumour; the CT angiography section localizing the distal osteotomy of the fibula; the chosen resection plane of the mandibular lesion; and the number of attempts required to correctly position and reposition both the cutting guides and the fibula flap. Prior exposure to immersive technologies was limited, with only four participants reporting previous experience, specifically half through recreational VR use and the other half through prior exposure to both VR and 3D printing in a medical setting.

#### VR environment

**Figure 1:**
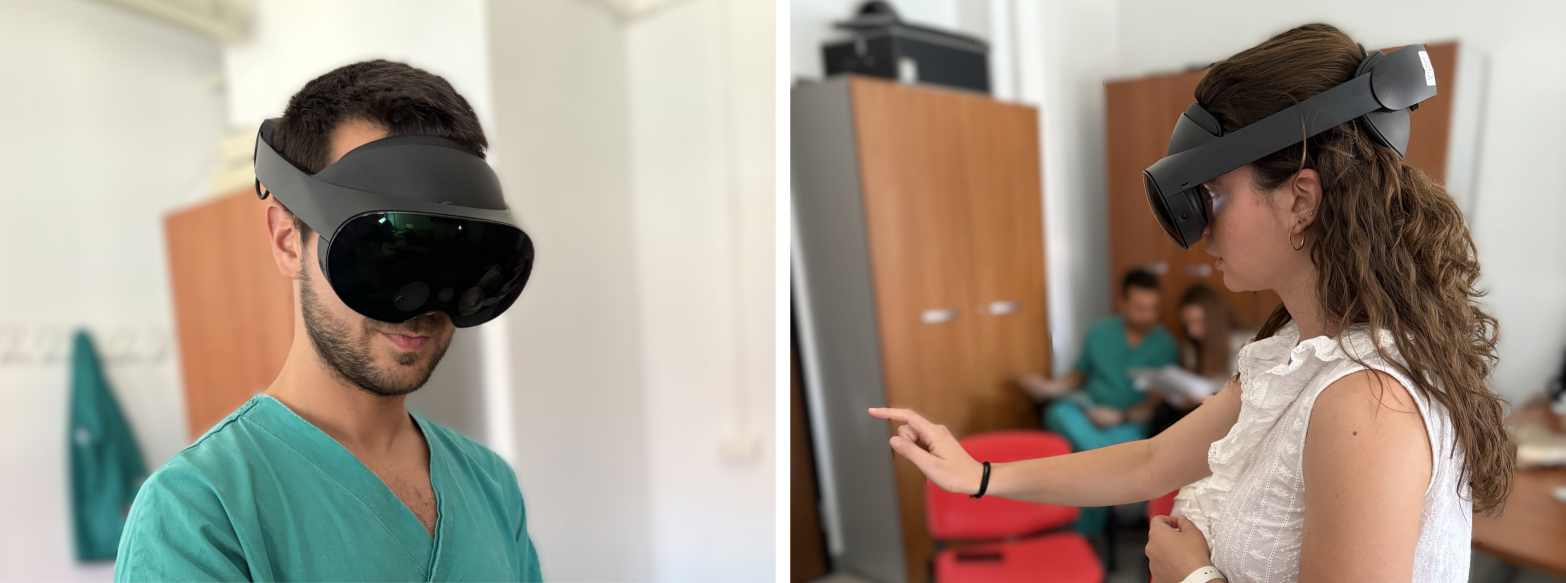
Two participants wearing the Meta Quest Pro headset while interacting with the VR environment.

The VR simulator was developed as a training platform for junior residents (**Figure 1**) in maxillofacial surgery to introduce the core concepts and spatial understanding required for mandibular reconstruction using a free fibula flap. Rather than aiming to replicate the complexity of a complete surgical procedure, the simulator focused on essential steps in the oncologic and reconstructive workflow, presented in a structured, interactive format suitable for early-stage learners. The Meta Quest Pro headset, powered by the Qualcomm Snapdragon XR2+ platform with a resolution of 1080x1920 pixels per eye, was used in this educational experience. All interactions in the simulator were conducted using a hand-tracking system, allowing users to manipulate and explore virtual objects with natural hand gestures, without the use of physical controllers. During the procedure, an operator maintained visual control on a tablet viewer of the environment in which the participant was carrying out the experience.

**Figure 2:**
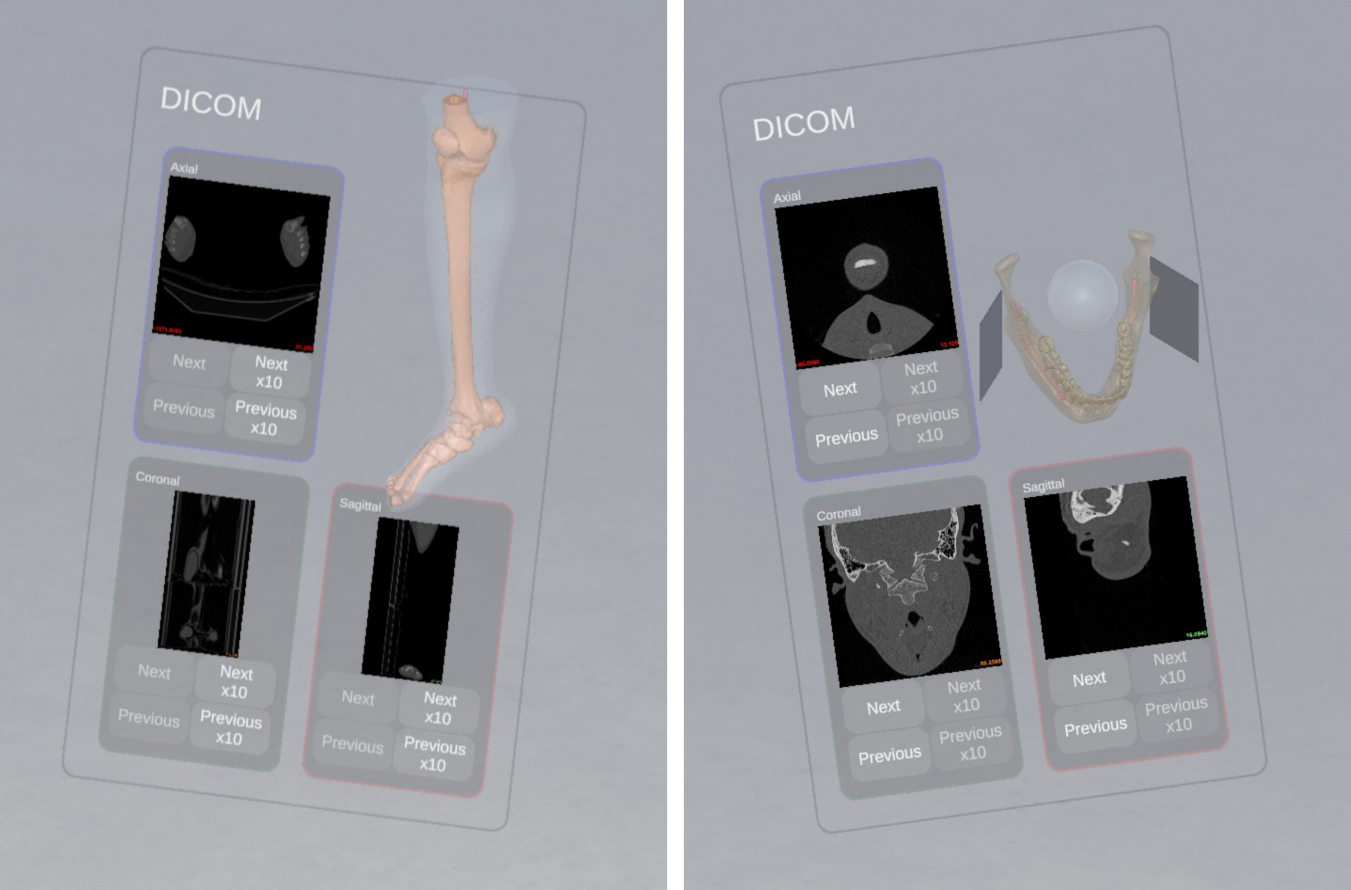
CT scans in axial, coronal, and sagittal plans and 3D model of the mandible (left) and the fibula (right).

Upon entering the VR environment, users were first introduced to the anatomical and radiological data of the case. DICOM files of the mandible affected by a tumour were available in both 2D (axial, coronal, and sagittal planes) and as a fully interactable 3D model. In addition, DICOM images from an angio-CT scan of the lower limb were provided to allow evaluation of the fibula and its vascular anatomy (**Figure 2**).

**Figure 3:**
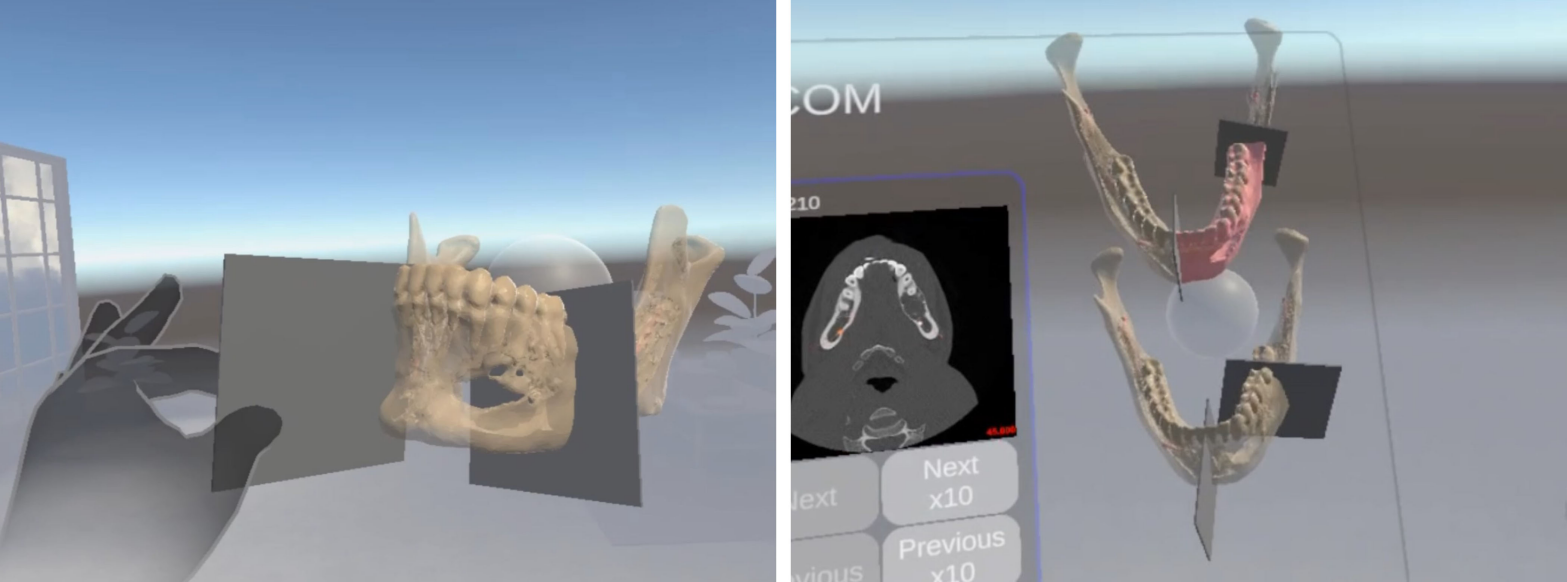
Osteotomy planes positioning. Osteotomies plans of mandible tumour defined by a participant in VR (left), and real-time comparison with participant and expert-defined surgical plan of osteotomy planes (right).

**Figure 4:**
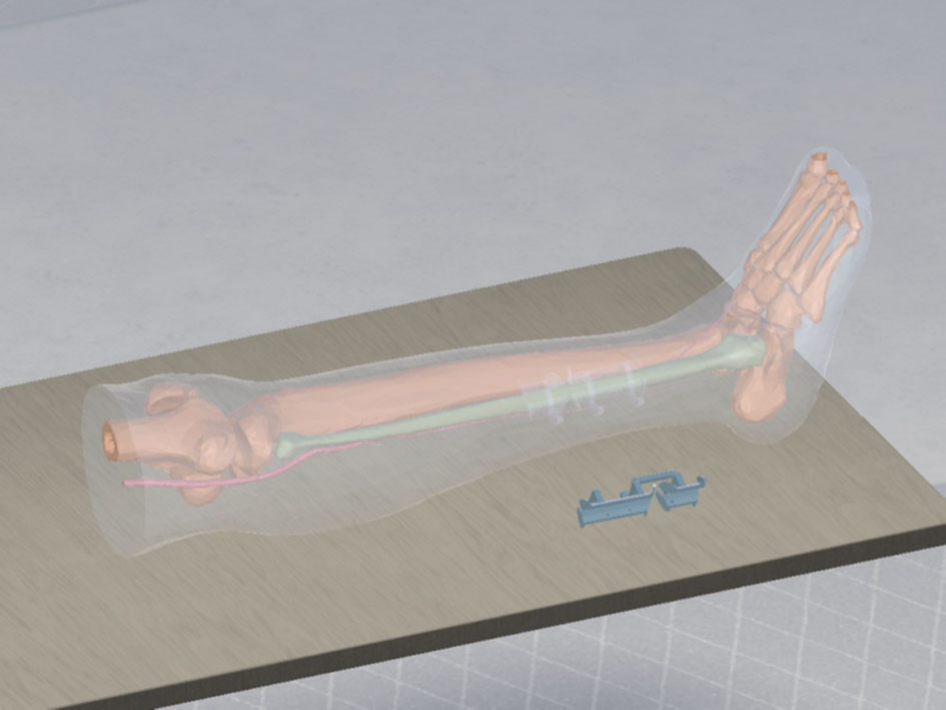
3D model of the fibula for position of cutting guide.

**Figure 5:**
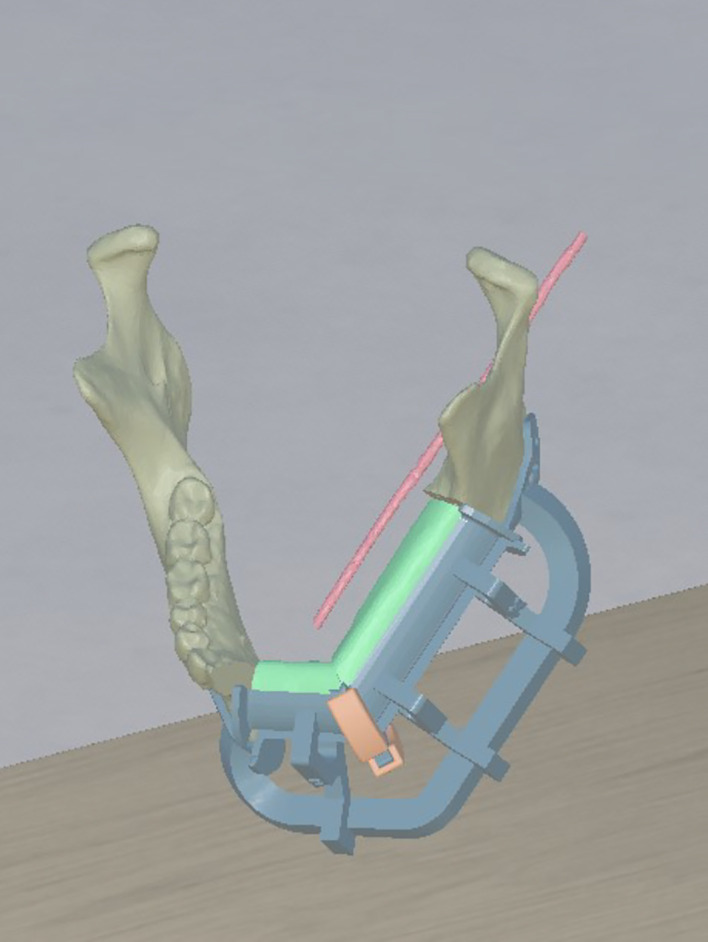
Virtual reconstruction of the mandible with fibula segments.

The first task required participants to identify tumour margins and define the appropriate osteotomy planes directly on the 3D model of the mandible. After confirming their choices, the system generated a real-time comparison with the expert-defined surgical plan (**Figure 3**), enabling trainees to assess the accuracy of their decisions and develop a deeper understanding of oncologic resection planning. In the following step, users interacted with a virtual 3D model of the fibula to choose one of three predefined positions for the cutting guide (**Figure 4**). While this does not reflect the complete flexibility of intraoperative decision-making, the availability of fixed options ensured consistency in training and allowed for standardized assessment across participants. The selected guide determined how the fibula segments would be harvested in the subsequent reconstruction phase. This immersive interface facilitated intuitive interaction, though some participants reported a learning curve, particularly during tasks requiring precise positioning. In the final part of the simulation, users virtually removed the tumour using the mandibular cutting guide and proceeded to reconstruct the defect using fibula segments (**Figure 5**). These segments were manually placed into the mandibular gap, following a predefined alignment intended to facilitate the task and ensure uniformity across trainees. This standardized setup emphasized the spatial reasoning involved in reconstructive planning while avoiding the technical barriers of complete surgical realism.

#### 3D printing

Exported DICOM from CT of a mandibular solid tumour and angio-CT of lower limbs data were segmented with Mimics Innovation Suite (Materialise). Slicing and printing were prepared with Preform (Formlabs), and models were printed on a Formlabs Form 3B (Formlabs Industries, Boston, USA) with a nozzle size of 0.4 mm, a slice thickness of 0.2 mm, and the printing transparent resin. Case was printed on a 1:1 scale.

**Figure 6:**
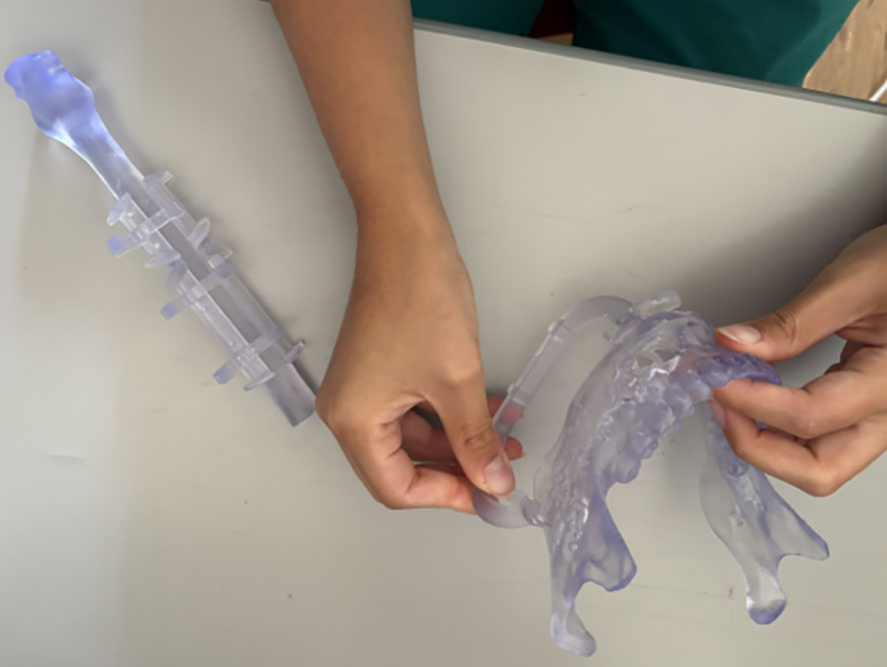
Participant interacting with 3D printed cutting guides and anatomical models.

Each participant interacted directly with the 3D printed models (**Figure 6**), following the key steps of the surgical simulation for mandibular reconstruction with a fibula free flap. After exploring the anatomical models, participants were asked to perform the guided osteotomies and to reposition the fibular segment within the mandibular defect using the printed cutting and repositioning guides. In addition, they were instructed to shape a custom titanium reconstruction plate to fit the defect, replicating the intraoperative workflow. Importantly, the 3D printed tasks mirrored the same surgical phases that participants had previously performed in the VR environment, ensuring methodological consistency between the virtual and physical simulations. For each resident, the total interaction time was recorded, along with the number of attempts required to correctly position the cutting guides and fibular flap. At the end of the session, participants were invited to provide structured feedback on their experience with the models and the simulation process.

### Questionnaire

After the experimental session, participants completed a questionnaire, including the System Usability Scale (SUS) (8) and the Net Promoter Score (NPS) (9) to assess usability and user satisfaction. The SUS is a standardized 10-item questionnaire providing a global measure of perceived system usability, with scores above 68 considered acceptable and those above 80 indicating excellent usability (10). The NPS quantifies user satisfaction and willingness to recommend the system, ranging from –100 to +100. According to the creators of NPS, Bain & Company (11), scores above 0 are considered good, above 20 favourable, above 50 excellent, and above 80 world-class. It is calculated using the following formula:


$$\begin{array}{l} NPS=(\frac{number\;of\;promoters-number\;of\;detractors}{total\;number\;of\;respondents})\times \\ 100=\;(\frac{8-0}{12})\times 100=67 \end{array}$$

where promoters are respondents who gave a score of 9 or 10, and detractors gave a score from 0 to 6 on the 0–10 likelihood-to-recommend scale.

Group comparisons for SUS and NPS were performed using the Kruskal–Wallis, a non-parametric method chosen due to the small sample size to determine whether the medians of the three groups (1^st^, 2^nd^, and 3^rd^ year) differ. To further explore the relationship between SUS and NPS scores, correlation was evaluated using Spearman’s rank correlation coefficient (r). Statistical significance was set at p < 0.05 (α).

## Results

The mean time to perform the procedure in VR was 16.10 minutes, compared to 11.50 minutes on 3D-printed models. When stratified by training year, first-year residents took slightly longer on average (18.25 min) than second-year (16.40 min) and third-year residents (13.60 min), although differences were not statistically significant.

Tumour localization varied among participants, with differences observed across training years. Three participants correctly identified the lesion in the same axial section and five in the same sagittal section, while coronal sections differed. Notably, third-year residents more consistently identified the mandibular resection plane within oncological safe margins, whereas first- and second-year residents showed greater variability. Four participants accurately positioned the proximal resection plane. Cutting guides were successfully positioned on the fibula after three attempts and on the mandible on the first attempt. The fibula segment was correctly set within the mandible reconstruction on the first attempt.

Regarding usability evaluation and satisfaction, the average SUS score across all participants was 74.6, indicating good usability. The mean NPS score was 67, indicating strong user satisfaction and willingness to recommend the experience. Indeed, most participants were promoters (8) rather than detractors (0), reflecting an above-average level of acceptance and indicating a strong positive perception of the VR system. SUS and NPS scores are reported in **Table 1**.

**Table 1:** System Usability Scale (SUS) and Net Promoter Score (NPS) reported for each participant.

Participant	Year	SUS	NPS
1	2^nd^	77.5	8
2	1^st^	52.5	7
3	1^st^	67.5	9
4	2^nd^	82.5	10
5	1^st^	70	10
6	2^nd^	57.5	7
7	2^nd^	90	10
8	1^st^	77.5	10
9	3^rd^	90	9
10	3^rd^	80	8
11	3^rd^	72.5	10
12	3^rd^	77.5	9
Total	–	74.6	67

**Figure 7:**
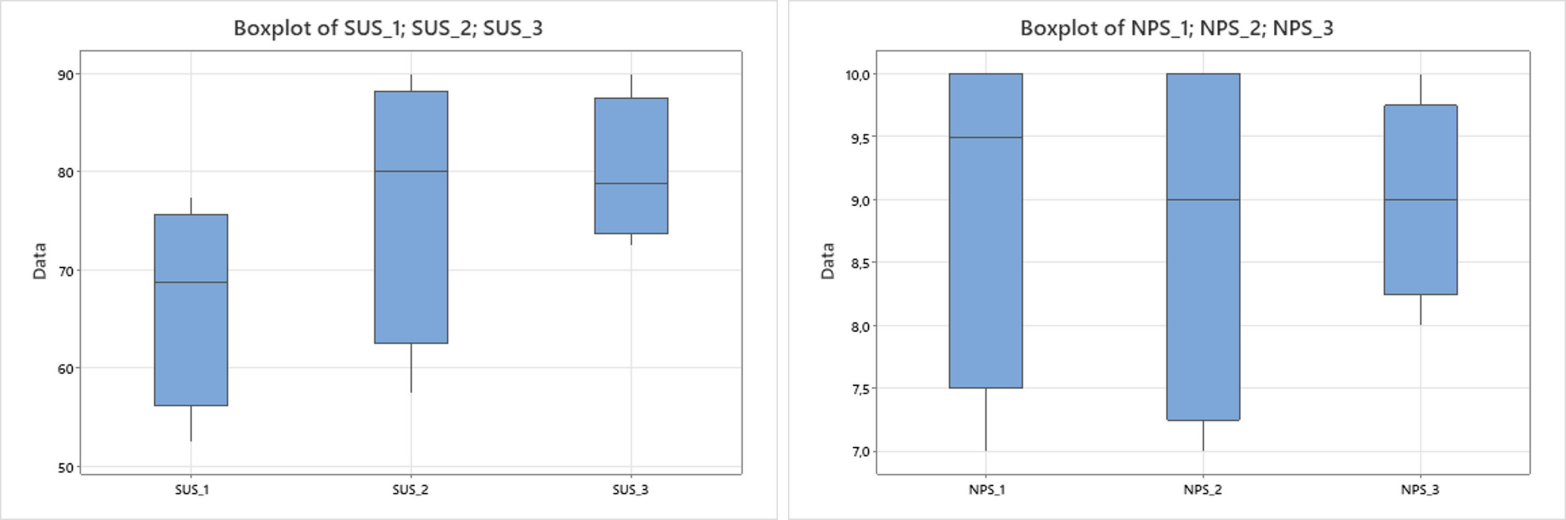
Boxplots showing the distribution of System Usability Scale (SUS) scores (left) and Net Promoter Score (NPS) values (right) across training years (first, second, and third). Mean SUS values show a progressive increase from first- to third-year residents, suggesting an improvement in perceived usability with growing surgical experience, while NPS scores remain relatively stable across groups, indicating consistently high satisfaction levels.

No significant differences were observed among years for SUS (p=0.166) or NPS (p=0.964). Although the results did not reach statistical significance, a progressive increase in mean SUS scores from first- to third-year residents was observed, as shown in **Figure 7** (left). In contrast, NPS values remained relatively stable across years, indicating consistent satisfaction levels regardless of training stage (**Figure 7**, right).

**Figure 8:**
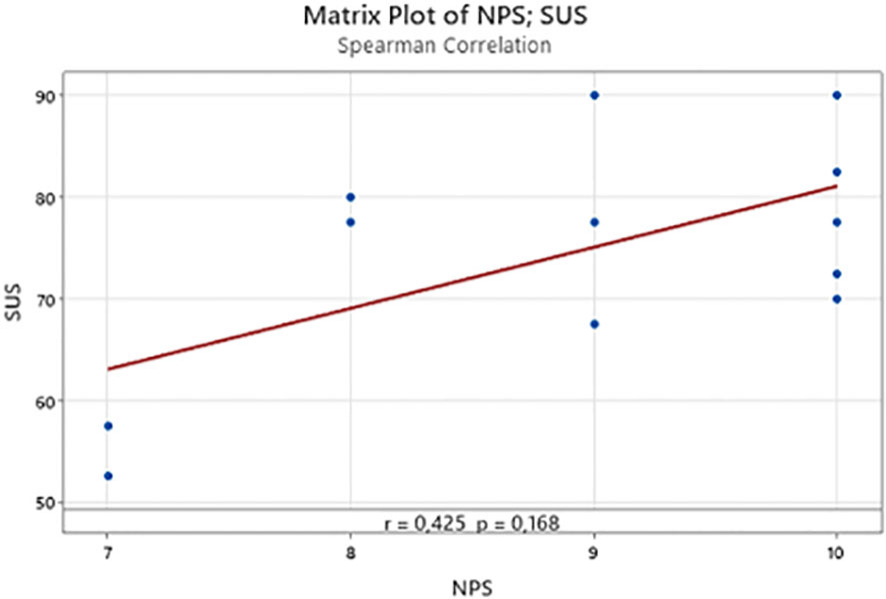
Scatterplot showing the correlation between System Usability Scale (SUS) and Net Promoter Score (NPS) across all participants. A moderate positive association was observed (Spearman’s r = 0.425, p = 0.168), indicating that higher perceived usability tended to correspond with greater user satisfaction and willingness to recommend the VR system.

To further explore the relationship between SUS and NPS scores, a Spearman correlation analysis (**Figure 8**) was computed. A moderate positive correlation was observed (r = 0.425), suggesting that participants who perceived higher usability tended to express higher willingness to recommend the VR system. However, the correlation did not reach statistical significance (p = 0.168), likely due to the small sample size. Visual inspection of the correlation plot shows a general upward trend, consistent with this moderate relationship, though with some dispersion reflecting individual differences in user perception and engagement.

Item-level analysis of the SUS further illuminated participant perceptions (**Table 2**). The statement “I think I would need the support of a technical person to be able to use this VR simulator” received the lowest score (12 out of 48), suggesting that several participants felt they would require assistance to operate the simulator independently, especially among first-year residents. Conversely, the highest-rated item was “I think I would like to use this system frequently” (42 out of 48), reflecting strong engagement and interest in continued use. All the other SUS items scored between 33 and 41, indicating a generally positive perception of usability. In particular, the SUS question “I thought the VR simulator was easy to use” scored relatively low (33 out of 48), ranking as the second-lowest item.

**Table 2:** System Usability Scale (SUS) questionnaire, reporting the sum and average for each item.

System Usability Scale	Sum	Average
I think that I would like to use this VR simulator frequently.	42	3.50
I found the VR simulator unnecessarily complex.	39	3.25
I thought the VR simulator was easy to use.	33	2.75
I think that I would need the support of a technical person to be able to use this VR simulator.	12	1.00
I found the various functions in this VR simulator were well-integrated.	39	3.25
I thought there was too much inconsistency in this VR simulator.	39	3.25
I would imagine that most medical residents would learn to use this VR simulator very quickly.	41	3.42
I found the VR simulator very cumbersome to use.	40	3.33
I felt very confident using the VR simulator.	35	2.92
I needed to learn a lot of things before I could get going with this VR simulator.	38	3.17

Beyond usability metrics, all participants rated their overall satisfaction with the VR experience at the maximum score (5 out of 5) on a Likert scale. They also acknowledged the educational value of integrating VR and 3D printing for oncologic and reconstructive maxillofacial surgery and identified potential applications in other subspecialties, including orthognathic surgery (10 participants), aesthetic/malformation surgery (3), and traumatology (7).

## Discussion

This study explored the combined use of VR and 3D printed anatomical models as educational tool for young residents in maxillofacial surgery, focusing on mandibular oncologic reconstruction. The results demonstrate that immersive VR, complemented by physical 3D printed guides, provides a safe, engaging, and reproducible training environment that was well received by participants, including those with limited prior exposure to digital planning technologies. The VR environment developed for this study will be made available upon request to encourage replication and further research.

Dalgarno and Lee (12) have identified several key factors that make VR effective as an educational tool, including representational fidelity, immediacy of control, and a sense of presence. Immersion refers to the objective characteristics of the virtual environment, whereas presence combines these objective aspects with the user’s subjective engagement in the virtual space. A responsive and realistic VR system, therefore, can reproduce the feeling of “being there,” even in simulated or remote environments.

Computer-aided design (CAD) and 3D printing techniques began to be adopted in the 1990s for the diagnosis and treatment of complex head and neck and maxillofacial pathologies. Literature reports that these technologies can improve diagnostic accuracy by nearly 30%, procedural precision by more than 35%, and reduce operative time by about 18% (13). Nowadays, 3D printing plays a key role in reconstructive surgery, especially in mandibular reconstruction (14, 15). Previous studies have demonstrated the utility of 3D printing for preoperative rehearsal, anatomical study, and production of patient-specific surgical guides (16, 17). Zavattero et al. (18) also highlighted the feasibility and cost-effectiveness of in-house 3D printing for both clinical and educational applications, enabling customized planning without relying solely on commercial providers.

In our study, usability and user satisfaction were rated positively, with a mean System Usability Scale (SUS) score of 74.6, consistent with good usability. The high Net Promoter Score (NPS) of 67 reflects strong user satisfaction and willingness to recommend the experience, highlighting its perceived value for surgical training. All participants reported maximum satisfaction (5/5) on a Likert scale, reinforcing the educational benefit of the platform despite some operational challenges. When compared with previous studies adopting VR within the medical field, our mean SUS score of 74.6 falls within the expected range for early-stage educational VR systems. Bhat et al. (19) designed a VR framework for interactive and immersive visualization of CBCT data to enhance the understanding of complex dental structures and pre-operative planning in dentistry. Their evaluation involving 12 medical experts demonstrated excellent usability, with a SUS score of 87. Similarly, Haque et al. (20) developed a VR gamified system to support hand exercises for stroke rehabilitation, reporting a SUS score of 85 among patients, relatives, and physiotherapists. In contrast, Hsiesh et al. (21) developed an immersive VR mirrored-hand system for upper-limb rehabilitation and reported a mean SUS score of 56.7, interpreted as only moderately acceptable usability and highlighting the need for improved user guidance.

In this context, our findings align well with the broader literature: the overall SUS score of 74.6 indicates good usability, consistent with effective and user-friendly VR applications, while the third-year residents achieved an average SUS of 80, reaching the threshold of excellent usability (10). Although statistical comparisons among training years did not reach significance, a progressive increase in SUS scores from first- to third-year residents was observed, suggesting that greater clinical experience and familiarity with digital planning tools may facilitate interaction with the VR environment. NPS values remained stable across training years, indicating consistent satisfaction levels regardless of experience. The moderate, though non-significant, positive correlation between SUS and NPS further suggests that higher perceived usability was generally associated with greater willingness to recommend the system. These findings reinforce that, while the system was well received overall, enhanced interface intuitiveness and user guidance could further optimize accessibility for less experienced users.

Item-level analysis of the SUS revealed areas for refinement and improvement. While most participants expressed enthusiasm for frequent use of the system, several indicated they would require technical support to operate it independently, especially first-year residents. The relatively low ratings for ease of use suggest that interface design and onboarding warrant further improvement, particularly for hand-tracking tasks such as positioning cutting guides on the mandible and fibula. These findings echo prior work showing that intuitive interactivity is essential for maintaining immersion and maximizing educational benefits in VR-based learning environments. Beyond usability, participants recognized the strong educational value of VR and 3D printing for oncological and reconstructive surgery, but also identified potential applications in orthognathic surgery, traumatology, and aesthetic or malformation surgery, underscoring the broader versatility of these technologies. This perception is consistent with literature describing the expanding role of VR and 3D printing in head and neck surgery, not only for preoperative planning but also for intraoperative guidance, patient communication, and multidisciplinary education (4).

From a pedagogical standpoint, this study aligns with experiential learning principles (7), which emphasizes that active, hands-on experiences lead to deeper understanding and longer retention compared with passive learning. By combining immersive visualization with tangible manipulation of anatomical models, VR and 3D printing bridge the gap between theoretical study and surgical practice. A recent review by Tene et al. (22) identified VR as a prevalent tool in medical education, highlighting a positive trend toward improved educational outcomes but also stressing the need for more rigorous evidence of efficacy. The findings of the present study support this direction, demonstrating the feasibility, safety, and strong acceptance of immersive technologies in maxillofacial surgical training.

Nevertheless, some limitations must be acknowledged. First, this was a single-center pilot study involving a limited number of participants. Initially, eight residents from the first and second years of the maxillofacial surgery program were enrolled, and the cohort was later expanded to include third-year residents, for a total of twelve participants. Although this number reflects the entire group available within the institution, it inevitably restricts statistical power and generalizability. Future multicentric studies including larger samples across different levels of surgical experience will be necessary to validate these findings.

Second, no control group was included. This choice was intentional, as the aim of the study was not to demonstrate superiority over conventional teaching methods, but rather to assess the feasibility, usability, and perceived educational value of VR and 3D printing as complementary learning tools. Traditional cadaveric dissection (23) remains the reference standard for surgical education; however, its use is limited by high costs, ethical considerations, and restricted accessibility. In this context, virtual and 3D printed simulations offer reproducible, cost-effective, and low-risk alternatives that can supplement rather than replace traditional training.

Third, the study primarily relied on subjective assessments of usability and satisfaction. Although these data provide valuable insight into user experience, objective performance metrics were limited. The time required to complete the procedure was recorded as a preliminary indicator of task efficiency, but future studies should incorporate additional quantitative measures, such as osteotomy accuracy, margin identification error, and flap alignment deviation, to better evaluate the educational and technical impact of these tools.

Another important limitation concerns model validation. The VR and 3D printed models were derived from clinical CT and CTA datasets used for actual patient-specific planning, ensuring high anatomical fidelity. However, a formal validation comparing these digital and physical models with intraoperative or cadaveric anatomical data was not performed. Such validation would be essential in future research to confirm dimensional precision and strengthen confidence in these educational simulators.

Finally, the development of a realistic and interactive VR environment required significant collaboration between clinicians and engineers. Although this initial phase was time- and resource-intensive, the resulting modules are reproducible and scalable, allowing for future integration into broader training curricula at a relatively low incremental cost.

In conclusion, this pilot study supports the integration of VR and 3D printing into surgical education as a means to enhance learning experiences and engagement among residents. Despite the small cohort and single-institution design, participants reported high satisfaction and recognized these technologies as valuable complements to traditional training. With further refinement, objective validation, and multicentre collaboration, immersive and 3D technologies hold significant potential to improve preparedness for complex procedures, optimize surgical planning, and ultimately contribute to better surgical outcomes.

## Data Availability

The raw data supporting the conclusions of this article will be made available by the authors, without undue reservation.
